# Blood Adhesion Molecules as Biomarkers in Children with Chronic Urticaria

**DOI:** 10.3390/children11040449

**Published:** 2024-04-08

**Authors:** Ioanna Angeli, Emilia Vassilopoulou, Dimitrios Cassimos, Ioannis Fotopoulos, Anastasios Serbis, Makis Alexandros, Sophia Tsabouri

**Affiliations:** 1 Department of Pediatrics, School of Medicine, University of Ioannina, 45332 Ioannina, Greece; angeli.joanne@gmail.com (I.A.); tsabouri@uoi.gr (S.T.); 2 Pediatric Unit, Fondazione IRCCS Ca’ Granda Ospedale Maggiore Policlinico, 20122 Milan, Italy; 3 Pediatric Department, Democritus University of Thrace, 68100 Alexandroupolis, Greece; dkasimos@med.duth.gr; 4 Department of Computer Science, University of Crete, Voutes Campus, 70013 Heraklion, Greece; ifotopoulos@csd.uoc.gr

**Keywords:** acute urticaria, chronic urticaria, childhood, vascular cell adhesion molecule 1, intercellular adhesion molecule 1

## Abstract

Background: The prevailing etiological model of both acute and chronic urticaria implicates specific allergen exposure that triggers the local release of vasoactive factors and inflammatory adhesion molecules, including vascular cell adhesion molecule 1 (VCAM-1), intercellular adhesion molecule 1 (ICAM-1), endothelial leukocyte adhesion molecule 1 (ELAM-1), P-selectin and E-selectin in the superficial dermis. This study focused on the possible role of VCAM-1 and ICAM-1 as biomarkers in children with acute and chronic urticaria. Methods: This study involved 184 children, 40 with acute urticaria, 71 with chronic urticaria, and 73 matched comparison subjects. The serum levels of ICAM-1 and VCAM-1 were determined in venous blood in all the participants on enrollment. Antihistamine treatment was administered to all the patients. In the children with chronic urticaria, the Urticaria Activity Score Questionnaire (UAS7) was completed daily by the parents. In 16 of the patients with acute urticaria and 43 with chronic urticaria, the serum levels of ICAM-1 and VCAM-1 were determined at follow-up after 6–8 weeks of treatment. Results: The mean serum levels of both VCAM-1 and ICAM-1 were higher in both groups of children with urticaria than in the comparison subjects at the start of the study. In the chronic urticaria group, the levels decreased significantly (*p* = 0.03 and *p* = 0.01, respectively) following treatment. Similarly, the acute urticaria group exhibited significant reduction in the mean levels of VCAM and ICAM (*p* < 0.001). In both groups, the mean level of ICAM after treatment was comparable with that of the comparison group. Conclusions: VCAM-1 and ICAM-1 are suggested as promising biomarkers for monitoring both acute and chronic urticaria in children. Future research should explore their utility in larger cohorts and investigate their role in personalized treatment strategies.

## 1. Introduction

Urticaria is a benign skin disorder characterized by pruritic, erythematous and edematous wheals [1,2,3]. The nature of the disorder is primarily autoimmune, and it usually presents soon (minutes–hours) after exposure to the responsible allergen. It is categorized into two main clinical types, chronic urticaria, in which the clinical symptoms tend to persist for long periods of time (more than 6 weeks) and/or relapse frequently over time, regardless of the treatment regimen [4,5], and acute urticaria (or acute spontaneous urticaria), the duration of which is much shorter (usually up to 4 h, but sometimes up to 24 h), which is more likely to evolve into the severe complication of urticarial vasculitis [1,6,7]. Chronic urticaria is commonly further classified into two subtypes: (a) chronic spontaneous urticaria, of idiopathic etiology, which represents the majority of reported cases, and (b) chronic inducible urticaria, which typically results from physical factors (e.g., tactile stimuli, pressure extremes) applied to the patient’s skin [7]. For the development and implementation of an optimal treatment plan tailored to each patient, particularly in children, clinicians must possess comprehensive knowledge of the primary pathophysiological mechanisms underlying the disorder. As indicated by current documentation, urticaria, whether acute or chronic, is attributed to the local release of histamine, bradykinin, kallikreins, prostaglandins, leukotrienes and other vasoactive factors from the mast cells and basophils in the superficial dermis [8,9,10].

These substances induce gradual or acute (depending on the clinical type of urticaria) capillary and venous vasodilation, leading ultimately to the clinically observed intradermal edema. Symptoms such as itching and a burning sensation are considered sequelae of the edema and typically do not necessitate specific clinical concern. Recent studies have identified various inflammatory biomarkers, including eosinophil cationic protein (ECP), tumor necrosis factor-alpha (TNF-α), complement, and a range of adhesion molecules, namely, intercellular adhesion molecule 1 (ICAM-1), vascular cell adhesion molecule 1 (VCAM-1), endothelial leukocyte adhesion molecule 1 (ELAM-1), P-selectin and E-selectin, which appear to contribute significantly to the molecular pathogenesis of urticaria [11,12,13,14,15]. The pathophysiological processes of urticaria, both immune-mediated (involving type I hypersensitivity reactions, autoimmune disorders) and non-immune-mediated (such as drug-induced non-allergic reactions or activation via strong emotional/physical stimuli), are well documented [16,17,18].

Regardless of the specific pathophysiological process, each case of urticaria is unique and requires a comprehensive medical history to clarify the precise causes in order to prescribe an appropriate treatment regimen. Typical treatment approaches include classic H1 antihistamines, newer histamine H2-receptor antagonists, leukotriene receptor antagonists and brief corticosteroid bursts, all of which have demonstrated significant clinical efficacy [19,20,21,22]. It is crucial to take into consideration serious drug safety concerns, especially in the case of children, who lack the legal, ethical, and mental-physical capacity to choose their treatment regimen.

In this context, the current study aimed to explore the utility of the specific adhesion molecules VCAM-1 and ICAM-1 as potential biomarkers for both acute and chronic urticaria in children. These adhesion molecules are known to play significant roles in a variety of physiological and pathological processes, including inflammation. VCAM-1 is a cell adhesion molecule that is expressed primarily on the surface of endothelial cells and plays a crucial role in the recruitment of leukocytes and other immune cells in the endothelium during inflammation. ICAM-1 is expressed in various cell types, including leukocytes and endothelial cells, and acts as a receptor for leukocyte integrins, facilitating their adhesion to the endothelium. The adhesion molecules selected for investigation were chosen because of their well-established causative role in urticaria pathogenesis and their accessibility via simple blood withdrawal. The primary objective of this study was to determine whether the two biological indices, VCAM-1 and ICAM-1, sampled from groups of children with urticaria, returned to normal levels after treatment. Success in achieving this goal would suggest effectiveness of the treatment for both chronic and acute urticaria and the efficacy of VCAM and ICAM as biomarkers.

## 2. Materials and Methods

### 2.1. Study Population

A total of 184 children, aged 1 to 16 years, who visited the Outpatient Department at the University Hospital of Ioannina during the period 2020–2022, were enrolled in this study. Of the 184 participants, 40 had been diagnosed with acute urticaria and 71 with chronic urticaria at the Outpatient Department of Paediatric Allergy, and 73 children were control subjects recruited from the Paediatric Clinic and the Outpatient Department of Paediatric Cardiology. The parents of all the children provided informed consent for them to participate in the study. The diagnostic assessment for all the patients with urticaria followed the guidelines of the European Academy of Allergology and Clinical Immunology (EAACI) and the World Allergy Organisation (WAO) and was carried out by two physicians who conducted the outpatient pediatric allergy clinic. The demographic and clinical characteristics of the children with urticaria in the study are shown in Table 1.

All the comparison subjects were age-matched (*p* < 0.05) with the patients with urticaria and had no type of acute or chronic illness, or any allergies that could affect the serum levels of VCAM-1 or ICAM-1. Patients presenting any type of induced urticaria, such as dermatographism or cholinergic urticaria, were excluded from this study. Before enrollment, it was confirmed that none of the participants, either patients or comparison subjects, had received any antihistamine treatment or montelukast. By excluding individuals with specific conditions and confirming the absence of certain medications, the study aimed to establish a better understanding of the role of VCAM and ICAM in the context of urticaria.

The study adhered to personal data protection legislation, and informed consent was obtained from a parent of each participant for the anonymous processing of their children’s data.

### 2.2. Assessment

All the participants underwent a comprehensive clinical and biochemical assessment according to the EEACI guidelines. The clinical examination comprised skin inspection, respiratory function and vital sign measurements. Allergy tests were performed, including radioallergosorbent (RAST) test and skin prick tests (SPTs). These tests help identify specific allergens that may contribute to the development of chronic urticaria. A differential blood count was conducted, and C-reactive protein (CRP) and erythrocyte sedimentation rate (ESR) were measured.

Extensive laboratory analysis was conducted to explore the etiology, triggers and mechanisms of chronic urticaria: measurement of serum IgE levels and markers of autoimmune diseases, including rheumatoid factor and anti-double-stranded DNA antibodies, to determine whether any autoimmune condition was associated with chronic urticaria. Tests for infectious diseases were conducted to rule out potential factors that could be triggering or exacerbating chronic urticaria, including serum parasite antibodies, stool assays for parasites, and the INFAI test to detect *Helicobacter pylori* infection. Evaluation for hereditary angioedema was performed by measurement of serum C1 esterase inhibitor.

Venous blood samples were collected from all participants at the beginning of the study, the patients with urticaria before the administration of any antihistamine treatment, and the comparison subjects to serve as a reference for the quantitative assessment of VCAM-1 and ICAM-1, and 6–8 weeks later, after the treatment, from those children receiving intervention with antihistamine treatment who attended for follow-up.

**Figure 1:**
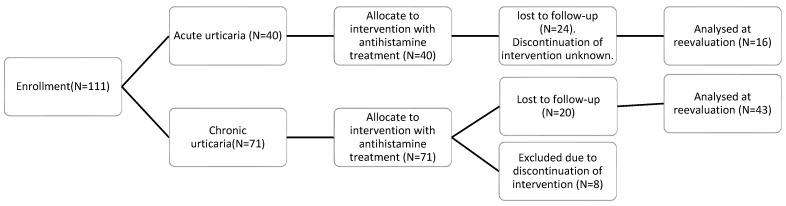
Flow chart of the study population of children with acute and chronic urticaria.

Of the 71 patients with chronic urticaria, only 51 attended follow-up evaluation, 43 of whom had adhered consistently to the prescribed antihistamine treatment. The patients with acute urticaria were also recommended antihistamine treatment. Of the 40 patients with acute urticaria initially advised to return for clinical reevaluation after 6–8 weeks, only 1 attended the follow-up visit and had been consistent with the prescribed antihistamine treatment. Thus, we compared all the patients at their first visit (N = 111) with the 73 comparison subjects, and with the patients who attended for reevaluation after 6–8 weeks of treatment, namely 16 patients with acute urticaria and 43 patients with chronic urticaria. The 8 patients with chronic urticaria who attended for reevaluation, but who had not received the antihistamine treatment prescribed, were excluded from the follow-up phase of the study (Flow Figure 1).

The patients diagnosed with chronic urticaria were advised to undergo antihistamine treatment according to the guidelines established by EAACI, as shown in Table 1. Specifically, second-generation non-sedating antihistamines such as levocetirizine and cetirizine, among others, were recommended in standard dosage, and 16.3% of the patients required a dose increase up to 2-fold from the initial dose. Only 9 of the patients (13.4%) received treatment with first-generation antihistamines, contrary to our recommendations. These patients took first-generation antihistamines because of the limited effectiveness on the symptoms of the second-generation antihistamines within the first 24 h of treatment, despite the parents having been clearly informed about the duration of treatment and the sometimes slow reduction of symptoms in the beginning. Consequently, the parents of these children resorted to using a first-generation antihistamine, either already stocked at home or prescribed by a physician.

Heparinized tubes were used for blood sampling, and the blood underwent centrifugation at 5 × 100 rpm for 5 min. The resulting plasma was separated into small tubes and stored at −15 °C until further analysis in the laboratory.

Quantitative determination of serum VCAM-1 and ICAM-1 was performed using a sandwich enzyme immunoassay technique with monoclonal antibodies. Reagents for these assays were sourced from R & D Systems (Biotechne, Minneapolis, MN, USA). The method exhibited a sensitivity of 0.156 ng/mL, with intra- and inter-assay coefficients of variations (CVs) of 1.95% and 7.8%, respectively.

The parents of the 71 children diagnosed with chronic urticaria were furnished with UAS7 questionnaires to systematically document the urticaria-related daily clinical symptomatology of the participants on a weekly basis. The questionnaire comprises two primary questions: the number of wheals observed on the patient’s skin and the intensity of pruritus experienced by the patient (qualitive). These questionnaires were diligently completed by the parents each day, enabling a thorough evaluation of urticaria activity and severity throughout the course of antihistamine treatment.

### 2.3. Statistical Analysis

Statistical analysis was conducted to determine the effect of the chosen treatment on the specific biological indices VCAM-1 and ICAM-1. The two-sample *t*-test with unequal variances, implemented in Excel, was employed for statistical assessment, with two-sided *p*-values calculated and a significance level set at *p* < 0.05.

## 3. Results

The analysis consisted of two parts. In the first part, we compared the mean serum levels of VCAM-1 and ICAM-1 between the comparison group (N = 73) and the patients (N = 111), of which 40 were experiencing acute symptoms (acute urticaria group) and 71 chronic symptoms (chronic urticaria group).

In the second part, we compared the mean serum levels of VCAM-1 and ICAM-1 between the control group and the patients with acute (N = 16) and chronic urticaria (N = 43) after 6–8 weeks of treatment with antihistamines.

**Figure 2:**
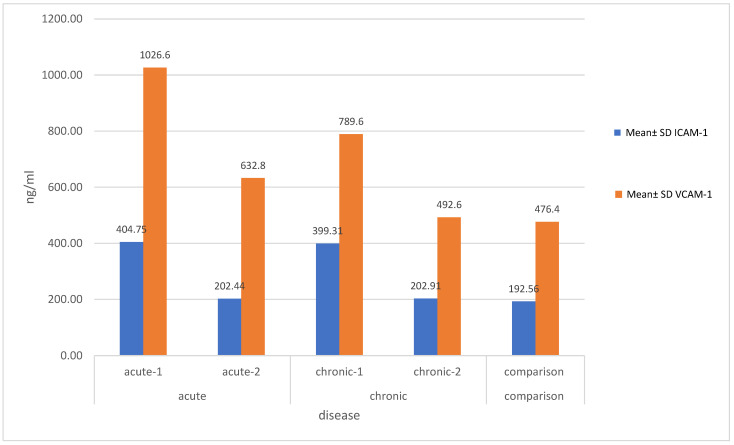
Bar chart showing the mean serum levels of vascular cell adhesion molecule (VCAM-1) and intercellular adhesion molecule (ICAM-1) in children with acute and chronic urticaria, before and after treatment, and in comparison subjects; acute-1: before treatment; acute-2: after treatment; chronic-1: before treatment; chronic-2: after treatment. SD: standard deviation; ICAM-1: intercellular adhesion molecule 1; VCAM-1: vascular cell adhesion molecule 1.

**Figure 3:**
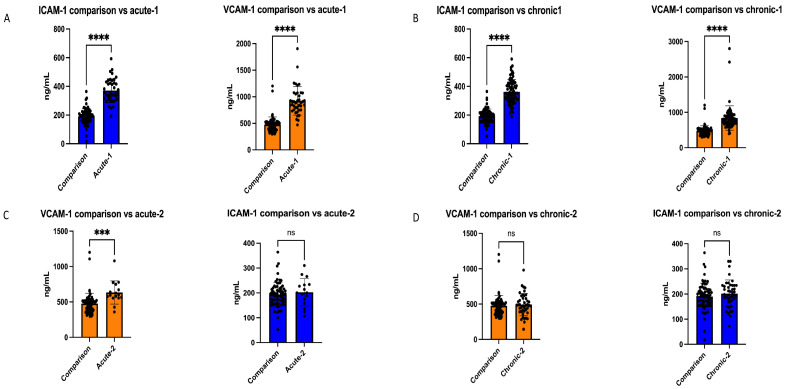
Scatter plot with bar presentation of the data: (**A**) serum levels of intercellular adhesion molecule-1 (ICAM) and vascular cell adhesion molecule-1 (VCAM) (mg/mL) in patients with acute urticaria before treatment (Acute-1) compared with the comparison subjects; (**B**) ICAM and VCAM in patients with chronic urticaria before treatment (Chronic-1) compared with the comparison subjects; (**C**) ICAM and VCAM in patients with acute urticaria after treatment (Acute-2) compared with the comparison subjects; (**D**) ICAM and VCAM in patients with chronic urticaria after treatment compared with the comparison subjects; *** *p*-value < 0,001; **** *p*-value < 0.0001; ns: not significant.

As shown in Figure 1, on initial measurement, the children with acute symptoms had mean serum levels of VCAM-1 of 1.026.6 ± 256.5 ng/mL and ICAM-1 of 404.75 ± 86.6 ng/mL, compared with 476.4 ± 143.1 ng/mL and 192.56 ± 48.91 ng/mL, respectively, in the comparison group. Correspondingly, the children with chronic urticaria had initial mean serum levels of VCAM-1 of 789.6 ± 158.1 ng/mL and ICAM-1 of 339.41 ± 76.5 ng/mL. A statistically significant difference was demonstrated in the mean levels of ICAM-1 and VCAM-1 between the children with both acute and chronic urticaria before treatment and the comparison group (*p* < 0.05, and *p* < 0.001, respectively), as shown in Figure 2 and Figure 3.

In the acute urticaria group, the mean serum level of VCAM-1 in the children who received treatment decreased to 632.8 ± 158.9 ng/mL following antihistamine treatment, with a statistically significant difference (*p* = 0.00), but remained higher than that of the comparison group. The mean serum ICAM-1 level in the acute urticaria group decreased to 202.44 ± 53.64 ng/mL following antihistamine treatment, a level comparable with that of the comparison group, and with a statistically significant change (*p* < 0.001) (Figure 2, Table 2).

In the chronic urticaria group, the mean serum level of VCAM-1 decreased significantly to 492.6 ± 155.9 ng/mL following antihistamine treatment (*p* = 0.03). Similarly, the mean serum level of ICAM-1 decreased significantly to 202.91 ± 55.47 ng/mL following antihistamine treatment (*p* = 0.01) (Figure 2, Table 2).

In the children with chronic urticaria who received treatment, the mean UAS7 score prior to antihistamine treatment was 23 (maximum score 42), while 6–8 weeks later, following antihistamine treatment, the score showed a statistically significant decrease, to a mean score of 7 (*p* < 0.0001).

## 4. Discussion

There is limited recent primary research investigating the role of VCAM-1 and ICAM-1 in urticaria, both in vivo and in vitro. Our study has provided valuable insight into the therapeutic management of chronic and acute urticaria in children, exploring the association between these adhesion molecules and urticaria and their changes in patients receiving treatment. The investigation of adhesion molecules in the context of urticaria holds promise for improving therapeutic approaches. Understanding the mechanisms by which these molecules are involved in the development and progression of urticaria could lead to the development of more targeted and effective forms of treatment and interventions. Recent reviews have discussed this issue and proposed the use of similar adhesion molecules for timely prognosis in urticaria [23,24].

Puxeddu and colleagues, in 2017, reviewed extensively the pathogenetic role of inflammation and angiogenesis mediators in chronic spontaneous urticaria. Their focus included the interleukine-1 (IL-1) family of cytokines, the IL-23/IL-17 axis, and TNF-α, emphasizing their involvement in chemokine signaling and leukocyte trafficking regulation. The authors also suggest potential biomarkers, such as prothrombin fragments 1  +  2, D-dimer, and CRP, highlighting the need for additional experimental work to validate these possible indices [24].

Two years later (2019), Puxeddu and colleagues continued their exploration of the clinical implications of biomarkers in urticaria, introducing additional potential indicators such as ESR and white blood count (WBC), and serum level of IL-6. They indicated the value of these markers for monitoring patients with urticaria, especially those with positive ASST, and suggested the prognostic role of various biomarkers related to angiogenesis, coagulation and vascular dysregulation, including vascular endothelial growth factor (VEGF), endostatin (ES), thrombospondin-1 (TSP-1), matrix metalloproteinase-9 (MMP-9), soluble VE-cadherin (sVE-cadherin) and the soluble forms of ICAM-1 and VCAM-1, the latter in agreement with our current findings [13].

In 2022, Raimondo and colleagues explored novel biomarkers for the immuno-pathogenesis of chronic inflammatory skin diseases, including urticaria. Their primary research, involving 13 patients with CSU and 10 healthy control subjects, demonstrated how dysbiosis of salivary microbiota may contribute to a dysregulated immune system in CSU. They proposed IL-18, soluble ICAM-1, and sE-selectin as potential biomarkers for use in the future. These findings provide validation for the clinical relevance of our study on ICAM-1 in the contribution to the current understanding of urticaria and its management.

In this study, we observed raised levels of ICAM-1 and VCAM-1 in children with both acute and chronic urticaria and a significant impact of antihistamine treatment on the levels of these adhesion molecules. Specifically, we demonstrated a significant decrease in the serum level of ICAM-1 following antihistamine treatment in both acute and chronic urticaria. The serum level of VCAM-1 exhibited a significant decrease in chronic urticaria, but the effect was comparatively less pronounced in acute urticaria. We were able to establish a correlation between the reduction in serum levels of these adhesion molecules and clinical improvement of urticaria symptoms. Our results demonstrated that the decrease in serum levels of ICAM-1 and VCAM-1 were correlated with an improvement in the symptomatology of the children, as indicated by the UAS-7 questionnaires completed by the parents. These observations underscore the potential of antihistamine treatment in modulation of the expression of adhesion molecules involved in the pathogenesis of urticaria. The significant reduction in the serum levels of ICAM-1 following treatment suggests a potential therapeutic target for both acute and chronic urticaria.

While our study offers valuable insight into the potential of VCAM-1 and ICAM-1 as prognostic biomarkers in acute and chronic urticaria in pediatric practice, it is essential to acknowledge its limitations. The relatively small sample size and the single-center approach might limit the generalizability of our findings. Additionally, the time frame for post-treatment evaluation, set at 6–8 weeks, may not capture longer-term variations in biomarker levels or clinical outcomes. We focused on VCAM-1 and ICAM-1, but parallel investigation of other possible biomarkers, which were not considered in this study, and their interplay in the pathophysiology of urticaria could contribute to a more comprehensive understanding. Further research with larger cohorts, longitudinal assessment and exploration of a wider array of biomarkers is warranted to validate and expand upon these preliminary findings.

In conclusion, our study indicates the potential utility of VCAM-1 and ICAM-1 as biomarkers in both acute and chronic urticaria in children. The significant decrease observed in these adhesion molecules after antihistamine treatment suggests their potential value in monitoring disease activity. These findings contribute to the growing body of knowledge, confirming the role of these adhesion molecules in urticaria pathogenesis. While preliminary, these results lay the groundwork for future investigations exploring a broader spectrum of biomarkers and their dynamic role in childhood urticaria, aimed at more refined diagnostic and therapeutic strategies.

## Figures and Tables

**Table 1:** Demographic and clinical characteristics and treatment of the study children with urticaria.

Demographic and Clinical Characteristics		Number (n = 111)	Percentage (%)
**Age groups (years)**	1–5	27	24.3
	6–10	51	45.9
	11–16	33	29.7
**Gender**	Male	57	51.3
	Female	54	48.7
**Disease**	Chronic urticaria	71	64
	Acute urticaria	40	36
**Symptoms**	Pruritus	87	78.3
	Wheals	100	90
	Angioedema	14	12.6
**Treatment received**	Levocetirizine	12	17.9
	Hydroxyzine	9	13.4
	Rupatadine	13	19.4
	Cetirizine	9	13.4
	Desloratadine	7	10.4
	Montelukast	3	3.2
	Ranitidine	3	4.4
	Methylprednisolone (intravenous)	12	14.9
	Dexamethazone (per os)	2	3

**Table 2:** Significance (*p*-values) of decrease in the mean serum levels of vascular cell adhesion molecule-1 (VCAM-1) and intercellular adhesion molecule-1 (ICAM-1) in children with acute and chronic urticaria after 6–8 weeks of antihistamine treatment (two-sample *t*-test analysis of variance).

**Biomarker**	acute urticaria (N = 16) vs. comparison (N = 71) before treatment	acute urticaria (N = 16) vs. comparison (N = 71) after treatment	chronic urticaria (N = 42) vs. comparison (N = 71) before treatment	chronic urticaria (N = 42) vs. comparison (N = 71) after treatment
**ICAM-1** (ng/mL)	<0.001	0.518	<0.001	0.32
**VCAM-1** (ng/mL)	<0.001	<0.01	<0.001	0.58
	acute urticaria (N = 16) vs. comparison (N = 71) before treatment	acute urticaria (N = 16) vs. comparison (N = 71) after treatment	chronic urticaria (N = 42) vs. comparison (N = 71) before treatment	chronic urticaria (N = 42) vs. comparison (N = 71) after treatment
**ICAM-1** (ng/mL)	*p* < 0.001	*p* = 0.518	*p* < 0.001	*p* = 0.32
**VCAM-1** (ng/mL)	*p* < 0.001	*p* < 0.01	*p* < 0.001	*p* = 0.58

## Data Availability

Data available on request due to restrictions. The data presented in this study are available on request from the corresponding author.
